# Surgical outcomes after instrumented lumbar surgery in patients of eighty years of age and older

**DOI:** 10.1186/s12891-016-1239-9

**Published:** 2016-09-22

**Authors:** Jen-Chung Liao, Ping-Yeh Chiu, Wen-Jer Chen, Lih-Hui Chen, Chi-Chien Niu

**Affiliations:** Department of Orthopedic Surgery, Chang Gung Memorial Hospital, Bone and Joint Research Center, Chang Gung University, No._5, Fu-Shin Street, Kweishian, Taoyuan, 333 Taiwan

**Keywords:** Elderly, Degenerative lumbar spine, Instrumented fusion, Outcomes

## Abstract

**Background:**

In Taiwan, the life expectancy of an 80-year-old man is 88.4 years and the life expectancy of an 80-year-old woman is 89.8 years. Some of these people will develop symptomatic degenerative lumbar diseases that interfere with an active lifestyle. These older surgical candidates usually ask the surgeon whether it would be safe to undergo surgery. However, there is no literature assessing the outcomes of laminectomy, fusion and posterior fixation for degenerative lumbar diseases in patients older than 80 years. The purpose of this study was to report the surgical outcomes of patients 80 years of age and older who underwent spinal decompression and instrumented lumbar arthrodesis for degeneration lumbar diseases.

**Methods:**

We retrospectively reviewed patients with degenerative lumbar diseases and spinal stenosis who underwent surgery between January 2010 and December 2012. Inclusion criteria were age greater than or equal to 80 years, decompression with instrumented lumbar arthrodesis, and at least 2 years of follow-up. Totally 89 patients were studies. Clinical outcomes were evaluated according to the Oswestry Disability Index (ODI) and visual analogue scale (VAS) of leg and back pain. Plain radiographs (lateral, anteroposterior, and flexion-extension) were used to assess the status of fusion and implant-related complications. Every complication during admission and any implant-related or failed-back syndrome requiring a second surgery was documented. *T* test and Fisher’s exact test were used for statistical analysis.

**Results:**

Five patients were lost to follow-up, and another 12 died during the follow-up period. One patient died due to cerebral stroke just 2 days after surgery, and the other 11 patients passed away 3 months to 4 years postoperatively. In all, 72 patients had an adequate follow-up: 44 were female and 28 were male. The average age at surgery was 82.5 ± 2.6 years (80 to 93); 63 patients underwent their first lumbar surgery, and nine patients received a second surgery. Patients underwent arthrodesis surgeries were from a single-level to a 7–level. Four patients developed complications (5.6 %, 4/72). At the final follow-up, the average ODI score was lower than the preoperative score (30.0 vs. 61.8) (*p* < 0.001). The average VAS score also showed improvement (leg: *p* < 0.001; back: *p* < 0.001). Forty-three patients were classified as “satisfied”, and 29 were “dissatisfied”. Longer operation time (*p* = 0.014) and development of complications (*p* = 0.049) were related to poor clinical results.

Radiographic follow-up showed that 53 patients had solid union, ten had a probable union, and nine had pseudarthrosis. More surgical segments led to a greater chance of pseudarthrosis (2.0 ± 0.9 vs 3.0 ± 1.8, *p* = 0.003).

**Conclusion:**

Longer instrumented segments and development of complications contributed to worse clinical and radiographic outcomes. With proper patient selection, posterior decompression with instrumented fusion can be safe and effective for patients 80 years of age and older with degenerative lumbar conditions.

## Background

Degenerative lumbar diseases, collectively known as the so-called “aging spine”, are secondary to degenerative osteoarthritis of the disc and facet joints of the involved segments, and usually result in considerable disability among the elderly. The World Health Organization defines older people as those aged > = 65 years [1]. However, advances in medical care and improvements in public health have resulted in a rapidly growing group of geriatric patients who continue to lead active lives well into their eighth and ninth decade. The 2011 data of the Ministry of the Interior in Taiwan reveal that the average lifespan was 79.1 years (male 76.0 years; female 82.5 years), and that one half of all males can live longer than 79 years and half of females can live to over 85 years old. The life expectancy of an 80-year-old male was 88.4 years and the life expectancy of an 80-year-old female was 89.8 years [2]. In 2050, the estimated life expectancy of the global population will rise to 86.6 years for females and 81.1 years for males [3]. Some of these aging people will develop symptomatic degenerative lumbar diseases that might fail conservative treatment and interfere with an active lifestyle. Since these aging people generally have a comorbid osteoporotic spine, they usually ask the surgeon whether it would be safe to undergo surgery. Early studies found that osteoporosis-related complications such as pseudarthrosis and screw loosening were increased in patients older than 65 years [4, 5]. With proper patient selection, however, those 65 years and older can expect a substantial improvement in their health-related quality of life after surgical decompression and arthrodesis of the lumbar spine [6, 7]. To date, there is little data in the literature to guide surgeons and patients over 80 years of age who are considering surgical treatment for their degenerative lumbar diseases. The purpose of this study is to report on the surgical outcomes of patients 80 years and older who underwent spinal decompression and instrumented lumbar arthrodesis for degenerative lumbar diseases.

## Methods

After obtaining approval from the institutional review board, we retrospectively reviewed patients with degenerative lumbar diseases and spinal stenosis who underwent surgery between January 2010 and December 2012 at the Orthopedic Department of Chang Gung Memorial Hospital. Inclusion criteria were age greater than or equal to 80 years, decompression with instrumented lumbar arthrodesis, and at least 2 years of follow-up. The main diagnosis for surgery was degenerative lumbar disease, including degenerative spondylolisthesis, degenerative lumbar scoliosis, and chronic disc degeneration. We excluded those patients who underwent operation because of osteoporotic vertebral fractures, infection, or tumor. Demographic data including age, sex, body mass index (BMI), fused segments, operation time, estimated blood loss, length of hospital stay, perioperative complications, and postoperative complications of all study subjects were collected from medical records. The preoperative medical comorbidity was recorded by the weighted Charlson Comorbidity Index (CCI) [8]. We also focused on any incidence of revision surgeries related to implant or adjacent segment degeneration.

### Evaluation

#### Clinical assessment

Clinical outcomes were evaluated using the Oswestry Disability Index (ODI) and the visual analogue scale (VAS) of leg and back pain [9, 10]. At our department, all patients planning to undergo spinal surgery would be asked to fill out pre-operative ODI, VAS of the leg, and VAS of the back questionnaires during admission; the final ODI and VAS questionnaires were completed in the outpatient department or by mail. Three types of ODI and VAS scores were obtained: pre-operative, final, and difference. The ODI difference means the final ODI scores are subtracted from the pre-operative ODI scores. The VAS difference means the pre-operative VAS scores minus the final VAS scores. We used percentage of ODI improvement as another index of clinical outcomes. The definition of percentage of ODI improvement was the ODI difference/pre-operative ODI.

#### Radiographic assessment

Plain radiographs (lateral, anteroposterior, and flexion-extension) were used to assess status of fusion and implant-related complications. Solid fusion was defined as visible; a continuing bridging fusion mass at the bilateral transverse processes and no motion in flexion-extension on stress radiographs. Probable fusion was defined as unclear bony trabecular continuity with no radiolucent interruption or motion in stress radiographs. Pseudarthrosis was defined as radiolucent interruption of the fusion mass.

At the final follow-up, patients were classified into “satisfied” and dissatisfied” groups: when patients’ ODI improvement > = 50 %, they were considered “satisfied”; when their ODI improvement < 50 %, they were categorized as “dissatisfied”. Patients were also grouped as “solid union” or “non-solid union”, based on radiographic outcomes. Pseudarthrosis and probable fusion were considered “non-solid union”.

#### Statistical analysis

Data were analyzed using the SPSS statistical software package (version 18.0; SPSS, Chicago, IL, USA). Continuous variables were presented as means + − standard deviation. The ODI and VAS scores were compared preoperatively and at the final follow-up using paired *t*-test. Continuous variables including age, BMI, ODI, VAS, operation time, blood loos, number of complications, numbness of solid fusion, surgical levels, and CCI between satisfied and dis-satisfied group were compared by independent *t*-test. Other categorical variables between satisfied and dis-satisfied group were compared using Fisher’s exact test. A two-tailed value of *p* < 0.05 was considered statistically significant.

## Results

From January 2010 to December 2012, 89 patients with degenerative lumbar diseases underwent lumbar instrumented fusion at our department. Twelve patients died during the follow-up period: one died 2 days after surgery because of cerebral vascular accident during admission, and the other 11 patients died after discharge (Table 1). Five other patients were excluded from the study because of inadequate medical data or they were lost to follow-up without final clinical outcomes. In all, 72 patients were enrolled into the current study: 28 males (38.9 %) and 44 females (61.1 %) with a mean age of 82.5 ± 2.6 years (range 80–93). The main diagnoses for surgery included degenerative spondylolisthesis (45/72, 62.5 %), degenerative lumbar scoliosis (18/72, 25 %), and adjacent spinal instability (9/72, 12.5 %). Sixty-three patients underwent their first lumbar surgery, and nine received revision surgery. The average number of arthrodesis segments was 2.3 ± 1.3 (range 1–7): 21 patients underwent a single-level arthrodesis, 28 underwent a 2-level arthrodesis, 14 had a 3-level arthrodesis, five had a 4-level arthrodesis, 2 had a 5-level arthrodesis, and two had a 7-level arthrodesis. The mean co-morbidities among these 72 patients were 1.6 ± 1.0, and the mean CCI was 1.77 ± 1.63. During admission, five patients had complications: one had a cerebral vascular accident, one had an implant loosening, two had pneumonia, and one had a urinary tract infection. During the follow-up period, delayed wound infection developed in one patient, and wound debridement was arranged for him. The other four patients had implant-related complications: three underwent revision surgeries at our hospital, and one went to another hospital for further help. The overall complication rate was 11.2 %.

**Table 1:** Causes of death during follow-up period

Etiology	Number of occurrence
Myocardial infarction	2
Sepsis	2
Pneumonia	2
Cerebral stroke	2
Lung cancer	1
Lymphoma	1
Colon cancer	1
Multiple myeloma	1

There was a statistically significant improvement in clinical measures (VAS and ODI) from the pre-operative to the final postoperative evaluation. The average VAS back scores improved from 6.3 ± 2.5 to 2.4 ± 2.3 (*p* < 0.001), and the average VAS leg scores improved from 5.2 ± 3.1 to 1.9 ± 2.6 9 (*p* < 0.001). The mean ODI improved from 61.8 ± 8.9 preoperatively to 30.0 ± 11.9 at the final evaluation (*p* < 0.001). The average ODI difference was 31.8 ± 12.5. The mean percentage of ODI improvement was 51.9 % ± 34.6 %; 43 patients had ODI improvement equal or over 50 %, and 29 patients had ODI improvement of less 50 %. At the final follow-up, the radiographs of 53 patients showed solid union, ten had probable union, and nine had pseudarthrosis.

### Analysis of predictive factors for “dissatisfied” and “non-solid union” results

We attempted to determine factors that were predictive of “dissatisfied” or “non-solid union” results. Of the 72 patients, 43 were classified as “satisfied” (ODI improvement > = 50 %), and 29 as “dissatisfied” (ODI improvement < 50 %). Fifty-three patients were categorized into the “solid union” group, and 19 into the “non-solid union” group. Factors such as age, sex, BMI, fused segments, preoperative ODI and VAS scores, preoperative CCI score, presence of complications, primary/secondary surgery, operation time, and blood loss were analyzed. When factors were compared between the satisfied and dissatisfied groups, patients with a complication and longer operation time were more likely to be dissatisfied (*p* = 0.014, *p* = 0.049), as were patients with longer fused segments (*p* = 0.053) (Table 2). A comparison of factors between the solid union and non-solid union groups (Table 3), revealed that longer fused segments (> = 3 segments) were more likely to result in a non-solid union (*p* = 0.003) (Table 3).

**Table 2:** Analysis between “satisfied” and “dis-satisfied” patients

	Satisfied group *N* = 43	Dis-satisfied group *N* = 29	*P* value
Sex (M:F)	16:27	12:17	0.722
Op age (years)	82.4 ± 2.5	82.6 ± 2.9	0.739
BMI	25.0 ± 3.2	25.9 ± 3.4	0.262
Pre-op VAS (back)	6.1 ± 2.4	6.4 ± 2.8	0.657
Final VAS (back)	1.4 ± 1.6	3.9 ± 2.5	<0.001
Pre-op VAS (leg)	4.9 ± 2.9	5.7 ± 3.3	0.259
Final VAS (leg)	0.8 ± 1.4	3.6 ± 3.0	<0.001
Pre-op ODI	60.6 ± 9.1	63.5 ± 8.7	0.500
Final ODI	15.0 ± 6.0	52.1 ± 9.6	<0.001
ODI difference	45.9 ± 10.3	11.4 ± 7.5	<0.001
Solid fusion (yes : no)	33:10	20:9	0.463
Primary: secondary surgery	38:5	25:4	0.785
Complication (yes : no)	2:41	7:22	0.014
Instrumented segment	2.0 ± 0.9	2.6 ± 1.7	0.053
Op time (minutes)	188.8 ± 45.0	214.1 ± 62.6	0.049
Blood loss (ml)	718.6 ± 572.8	903.1 ± 685.7	0.220
CCI	1.7 ± 1.8	1.8 ± 1.4	0.834

*M* male, *F* female, *Op* operation, *BMI* body mass index, *VAS* visual analog scale, *ODI* oswestry disability index, *CCI* the Charlson comorbidity index

**Table 3:** Comparisons between patients with solid fusion and not solid fusion

	Solid fusion *N* = 53	Not solid fusion *N* = 19	*P* value
Sex (M:F)	21:32	7:12	0.831
OP age (years)	82.5 ± 2.5	82.3 ± 3.0	0.786
BMI	25.0 ± 3.5	26.1 ± 2.6	0.189
Pre-op VAS (back)	6.3 ± 2.7	6.2 ± 2.3	0.938
Final VAS (back)	2.5 ± 2.5	2.0 ± 2.0	0.356
Pre-op VAS (leg)	5.5 ± 3.1	4.4 ± 2.9	0.188
Final VAS (leg)	2.0 ± 2.7	1.7 ± 2.2	0.705
Pre-op ODI	62.5 ± 9.0	59.7 ± 8.9	0.554
Final ODI	30.0 ± 12.4	29.8 ± 10.9	0.979
ODI difference (final - pre-op)	32.6 ± 13.1	29.9 ± 11.2	0.689
ODI improvement (%)	0.5 ± 0.4	0.5 ± 0.3	0.612
Primary : secondary surgery	46:7	17:2	0.762
Complication (yes : no)	7:46	1:18	0.344
Instrumented segment	2.0 ± 0.94	3.0 ± 1.8	0.003
Op time (minutes)	194.4 ± 51.0	211.9 ± 60.7	0.226
Blood loss (ml)	714.3 ± 530.9	1012.1 ± 804.2	0.074
CCI	1.6 ± 1.6	2.3 ± 2.2	0.127

*M* male, *F* female, *Op* operation, *BMI* body mass index, *VAS* visual analog scale, *ODI* oswestry disability index, *CCI* the Charlson comorbidity index

## Discussion

The definition of “elderly patients” who underwent spinal decompression with instrumentation used in the spine literature is inconsistent. At the beginning of the 21st century, some spine surgeons set 65 years of age as a cut-off to study elderly patients [11], others used 70 years of age as a cut-off point [12–14]. Wu et al. reported 82 patients with degenerative spondylolisthesis aged 65 and older who underwent posterior instrumented fusion after a 2-year minimum follow-up, the average ODI score improved from 56 preoperatively to 32 finally; almost 75 % of patients could achieve solid fusion [11]. They concluded that a higher preoperative ODI score might lead to a dis-satisfied result, and lower bone mineral density was a risk factor for non-solid fusion. Glassman studied 50 patients 65 years of age and older who underwent a single-level posterolateral lumbar arthrodesis. The results showed a mean improvement in ODI scores of 28.5 points; the mean improvement in short-form 36 (SF-36) scores was 14.2 points. The total numeric rating scale for back and leg pain also showed a 10.4-point improvement at the 2-year follow-up. In 2003, Ragab et al. published the first report using 70 years of age as a cut-off for elderly patients undergoing lumbar surgery [13]; although overall morbidity was 20 %, the final satisfaction rate was as high as 92 %. Okuda et al. studied 101 patients with L4-L5 degenerative spondylolisthesis that underwent posterior lumbar interbody fusion with pedicle screws [14]. Group 1 had 31 patients aged over 70 years, and group 2 had 70 patients aged 70 years; although collapsed union and delayed union were more common in the elderly group, the clinical results were similar without a difference. Becker et al. used the VAS, ODI and SF-36 to analyze the clinical results of 195 patients aged 70–89 years who underwent lumbar spinal fusion. VAS back and leg pain were initially reduced by >50 %, and the average ODI and SF-36 were improved, so they concluded that age itself cannot be considered a contraindication [12]. In recent years, the cut-off age was increased to 75 years. Crawford et al. studied 11 men and 24 women with a mean age of 78.3 years (range 75–85) that underwent posterolateral lumbar arthrodesis: the health-related quality of life measures, including the VAS, ODI and SF-36, were improved significantly from pre-operative to 2-year postoperative [15]. Costa et al. evaluated the clinical and radiographic results of 53 patients at a mean age of 77.8 years (range 75–82) that underwent lumbar instrumented fusion. The preoperative VAS was 7.8, and improved to 4.1 at the 18-month follow-up; the ODI also had a 25.9 % improvement at the last evaluation. Stable fusion was observed in 78 % of patients. Nine patients (17 %) presented surgery-related complications [16]. In the present study, we set the cut-age of the research at 80 years because there are more and more patients older than 80 years old who are candidates for instrumented lumbar surgeries in our country. Similar to clinical results of younger patients (60 years-old or 70 years-old) list above, most patients with 80 years or older in this study could obtain a certain degree of symptom relief by ODI or VAS score assessment. And 59.7 % (43/72) patients were totally satisfactory to their surgical outcome at final follow up.

In the current study, we found longer instrumented fusion was more likely to result in non-solid fusion, which could lead to poor clinical results. We believe that if longer fusion levels were performed, there would be more blood loss, longer operation time, and more complications. However, whether the number of levels fused is associated with complications is still controversial. Daubs et al. reported that the number of levels fused was not a significant factor for complications [17]. However, Carreon et al. found that more fused levels corresponded with an increase in the prevalence of complications in patients 65 years of age or older with a mean number of fused levels of 2.4 [18]. Acosta Jr et al. found that patients 75 years of age and older who underwent thoracic and/or lumbar arthrodesis across five or more levels had a complication rate up to 62 % [19]. A report from Raffo et al., which studied 20 patients older than 80 years who underwent lumbar arthrodesis, showed that the number of levels fused had a strongly significant correlation to days spent in the intensive care unit, but the authors did not mention the relationship between the number of levels fused and complications or clinical results [20].

Decompression only for unstable spine might result in further instability with the sequel of severe back pain [21], therefore, fusion after decompression has been recommended for two decades. Zdeblick and Fischgrund et al. reported the clinical and radiographic superiority of arthrodesis with supplementary instrumentation [22, 23]. It also has been known that instrumentations increase the arthrodesis rate and enhance clinical outcome, even in the elderly [24]. However, there are two major concerns when performing spinal fusion surgery with pedicle instrumentation in the elderly. First, increasing numbers of co-morbidities in the elderly might result in high perioperative mortality or morbidity. Second, advanced osteoporosis in these elderly patients might result in pseudarthrosis and screw-related complications. In our current study, we could not find a significant effect for co-morbidity linked to dissatisfied results or pseudarthrosis rate, but the number of co-morbidities was really higher in the dissatisfied group (1.83 vs 1.49, *p* = 0.177). Raffo et al. demonstrated that in patients in their ninth decade, co-morbidity may predict major complications after undergoing lumbar spine arthrodesis, and suggested choosing patients with less preoperative co-morbidity to minimize complications [20].

Although osteoporosis-related complications such as a high pseudarthrosis rate or implant-related complications are expected in these advanced-age patients, only five patients (6.8 %) had screw-related complications in the current study, and the incidence of non-solid union was 26 % (19/72). Compared to previous reports on younger patients, this incidence was not high. Wu et al. reported a non-solid union rate of 24.3 % (20/82) in 82 patients aged over 65 years who underwent instrumented lumbar surgeries, and seven patients had implant-related complications (8.5 %) [11]. A historical study by Fischgrund et al. found that the non-solid fusion rate in patients at a mean age of 69 years and with one-level degenerative spondylolisthesis that underwent lumbar instrumented fusion was 18 % (6/35); 5.7 % had implant-related complications (2/35) [23]. Our results were similar to those of Wu et al. and Fischgrund et al. [11, 23], but the average age at surgery was older in our series (82.5 versus 69 years), which revealed that an age of 80 years was no longer a negative predictive factor for radiographic results or implant-related complications.

The present study does not identify a correlation between age over 80 years and operative complications; also does not reveal preoperative co-morbidities might be lead to a poorer outcomes, which does not mean we should encourage these old age patients to receive surgeries. To achieve good outcomes, patient selection is important. Spinal surgeons must evaluate the severity of patient symptoms, influence of these symptoms on quality of left, patient expectations, and willingness of the patient to take risk. Before surgery, any co-morbidity should be corrected or controlled to a stable condition. For those patients with high degree of pre-operative co-morbidities (usually a CCI greater than 5) or within unstable condition, we do not advocate operations.

## Conclusions

Our results showed that 74 % of patients who underwent lumbar instrumented fusion at an advanced age had satisfactory radiographic results and obtained improvement in ODI and VAS clinical outcomes. Perioperative mortality and complications were low. Age alone is not a contraindication for instrumented lumbar surgery. Number of fusion levels (over three segments) may be related to an increase in complications and have negative effects on final outcomes. To obtain good results, proper selection of patients at an advanced age remains a priority.

## Abbreviations

BMI: Body mass index
CCI: Charlson comorbidity index
ODI: Oswestry disability index
VAS: Visual analogue scale
